# The priorities, challenges, and scope of clinical communication teaching perceived by clinicians from different disciplines: a Hong Kong case study

**DOI:** 10.1186/s12875-022-01770-3

**Published:** 2022-06-22

**Authors:** Jack Pun

**Affiliations:** Department of English, City University of Hong Kong, 83 Tat Hong Avenue, Kowloon Tong, Hong Kong SAR, China

**Keywords:** Clinical communication, Multidisciplinarity, Interpretative phenomenological analysis (IPA), Medical educators, Hospital staff, Perceptions, Teaching

## Abstract

**Background:**

In the absence of a well-rounded syllabus that emphasises both interpersonal and medical dimensions in clinical communication, medical students in the early stages of their career may find it challenging to effectively communicate with patients, especially when dealing with perceived priorities and challenges across different disciplines.

**Methods:**

To explore the priorities, challenges, and scope of clinical communication teaching as perceived by clinicians from different clinical disciplines, we recruited nine medical educators, all experienced frontline clinicians, from eight disciplines across seven hospitals and two medical schools in Hong Kong. They were interviewed on their clinical communication teaching in the Hong Kong context, specifically its priorities, challenges, and scope. We then performed interpretative phenomenological analysis of the interview data.

**Results:**

The interview data revealed five themes related to the priorities, challenges, and scope of clinical communication teaching across a wide range of disciplines in the Hong Kong context, namely (1) empathising with patients; (2) using technology to teach both the medical and interpersonal dimensions of clinical communication; (3) shared decision-making with patients and their families: the influence of Chinese collectivism and cultural attitudes towards death; (4) interdisciplinary communication between medical departments; and (5) the role of language in clinician–patient communication.

**Conclusions:**

Coming from different clinical disciplines, the clinicians in this study approached the complex nature of clinical communication teaching in the Hong Kong context differently. The findings illustrate the need to teach clinical communication both specifically for a discipline as well as generically. This is particularly important in the intensive care unit, where clinicians from different departments frequently cooperate. This study also highlights how communication strategies, non-verbal social cues, and the understanding of clinical communication in the Hong Kong Chinese context operate differently from those in the West, because of differences in sociocultural factors such as family dynamics and hierarchical social structures. We recommend a dynamic teaching approach that uses role-playing tasks, scenario-based exercises, and similar activities to help medical students establish well-rounded clinical communication skills in preparation for their future clinical practice.

## Background

The teaching of clinical communication has been extensively explored in the field of medical education in the West, including with regard to curricula, assessment, learning outcomes, and educator training [1–7]. The recent expansion of this research area has been reflected in the increased emphasis on communication skills in many medical education programmes worldwide. Moreover, regardless of this emphasis in current teaching, clinicians and medical practitioners have to interact with patients from different milieus and with varied experiences. Therefore, knowing how to adapt to address patients’ concerns and communicative needs is of substantial importance for medical students [8].

Researchers have begun to conduct empirical studies to test the effectiveness of different teaching models. For example, Brown and Dearnaley investigated the effectiveness of an integrated clinical communication programme involving a medical school and a hospital [9]. They concluded that such an integrated approach could motivate students to reflect on the model of patient-centred care, its usefulness for collecting clinical histories, and its role in building rapport with patients. The study thus demonstrated that an integrated approach could help medical students bridge the gap between learned theory and practice by teaching them to approach clinical communication holistically. A quasi-experimental study conducted by Rashwan et al. further demonstrated the effectiveness of holistic medical training by evaluating the effects of scenario-based clinical simulation (SBCS) sessions on students, including their anxiety levels [10]. According to the findings, all of the students in the intervention group achieved satisfactory total percent scores when tested on their clinical communication skills after two weeks of SBCS sessions, while only 20% of the control group performed well (*p* < 0.001). It was also found that the first group of students were able to train their psychomotor, cognitive, and interpersonal skills simultaneously in one clinical scenario during a simulation session. The findings suggest that training such as SBCS sessions enables medical students to collaborate and reflect on each other’s performance, thus preparing them to work as teams in highly complex medical settings [11].

Experiential learning is another approach to clinical communication learning that helps students connect theories to real practice. Quilligan found that medical students could maximise their experiential learning in busy wards by practising their communication skills in a wide range of scenarios [12]. The findings also revealed medical students’ ability to adapt their communication based on the needs of an environment made complicated by the diversity of patient groups and their different health literacy levels, the nature of observed interactions, the students’ own actions, and input from role models. Experiential learning and holistic and integrated approaches to clinical communication teaching can thus help medical students gain practical and real-life experience in encountering patients. However, these methods might not be sufficient to help students learn how to deal with rare but critical clinical situations. As Shorey et al. noted, this is partly why medical educators use technological tools such as virtual reality platforms in modern pedagogy [13]. Using the Virtual Counselling Application using Artificial Intelligence developed by the National University of Singapore, the researchers found that the use of virtual patient simulations enhanced the students’ sense of preparedness and confidence, thus helping them become more effective communicators.

As most studies on clinical communication training have been conducted in the West, their findings may not apply to the socioculturally different East. In addition, they may have neglected developments in the latter context. Consequently, the perceptions of clinical communication and its training by Eastern physicians need to be explored and reported to improve clinical communication and thereby patient-centred care in the East. For instance, Ishikawa and Yamazaki outlined the ways in which studies may not have accounted for how clinical communication may differ across sociocultural contexts [14]. They based their compositional approach to culture on the idea that culture is a manifestation of all aspects of social life and is a layered structure of rituals, practices, hidden beliefs, and assumptions. They noted some core cultural differences between the East and the West that affect physician–patient relationships: (1) individualism vs collectivism and (2) high-context vs low-context communication. For example, as members of a high-context culture, Chinese people in general are evasive about death; some even believe that mentioning the word ‘death’ can lead to actual deaths [15]. This evasive attitude leads many patients’ families to misguidedly believe that modern medicine is always able to prevent deaths, and that death is due to the doctors’ failure to properly treat patients [15]. In this way, end-of-life communication in the Chinese context is very different from that in the West. Achieving effective patient-centred care requires research into the lived experiences of medical educators to explore the teaching of clinical communication in culturally specific contexts. This research would facilitate the development of local and culturally appropriate teaching models and cultural competence among students.

### Clinical communication in the East Asian and Hong Kong contexts

As noted by Lu et al., globalisation has made it necessary for medical educators to respond to increased cultural diversity [16]. Cultural competence, an attribute that has been stressed by the US Association of American Medical Colleges [17] and the UK General Medical Council [18], is important for medical professionals because it helps to prevent miscommunication and establish culturally appropriate expectations of healthcare [19]. The role that medical educators play in helping medical students prepare for effective communication with their future patients, regardless of the patients’ social or cultural backgrounds, has thus become widely recognised [20–22].

While clinical communication teaching as a whole has been investigated widely in recent years [20], the aforementioned recognition has in the past few decades led to a specific increased focus on cultural competence within this research. Within the international medical community, the capacity to communicate effectively with patients from specific cultural backgrounds has become commonly recognised as a desirable attribute of a graduate [23–26], and a comprehensive system for evaluating cultural competence in medical education has been developed [16]. However, there is a dearth of studies exploring this topic in the Chinese context. The lack of attention in this context to the diversity of patients’ cultural backgrounds also means that local curricula have not considered cultural competence to be a core element of medical professionalism [27].

A number of studies highlighting the particularities of East Asian clinical contexts have demonstrated the importance of filling this research gap and moving beyond strictly Western models of communication practices in clinical settings. For instance, in a review article on health professional–patient communication practices in East Asia, Pun et al. noted that patients from different East Asian countries have different attitudes towards death and terminal illnesses [28]. Specifically, Taiwanese patients’ families are often reluctant to discuss end-of-life issues. Upon receiving bad news, both Taiwanese and Korean patients’ families leave decision-making to the oldest family member. Japanese patients’ families tend to bring patients home for end-of-life care. By highlighting the role of families in decision-making on treatment, Pun et al. demonstrated the complexity and heterogeneity of clinician–patient communication in East Asia [28]. These findings indicate that when incorporating the teaching of cultural competence into medical education systems, it is necessary to develop culturally appropriate communication approaches for specific cultural contexts within Asia.

To teach clinical communication with cultural appropriateness in a specific Asian context, it is important to first break down the cultural homogeneity inherent in the existing medical education system. In Hong Kong, for example, there are two official languages, namely Chinese (both written Chinese and its spoken varieties, including Cantonese and Putonghua) and English, which leads to difficulties specific to medical practice in the region [29]. In a study examining clinical handover in a bilingual setting in Hong Kong, Pun found that the bilingual staff usually had little to no familiarity with Chinese medical terminology and thus read and recorded almost all of the medical information in written English [30]. Pun also observed that most of the staff in this bilingual context code-switched or engaged in translanguaging between spoken Cantonese and English medical terminologies [31]. These findings relate to the problem of miscommunication that may arise due to differences in the language used for medical terms and everyday conversation, as indicated in Pun et al.’s earlier study, which observed that medical information was altered when staff switched between spoken Cantonese and spoken English [32]. The findings of these studies indicate that for medical students, the homogenous use of English in the teaching of clinical communication leads to gaps in their training, potentially leading to miscommunication with local Cantonese-speaking patients in real-life practice.

Beyond linguistic concerns, research has found that doctors in Chinese-speaking Asian contexts rely on their own experiences rather than formal medical education to learn about and adapt to different cultural contexts [16]. In a study conducted in Taiwan, Lu et al. found that the lack of teaching materials on cultural competence in clinical communication had led to culturally essentialist beliefs, such that Taiwanese doctors tended to stereotype and oversimplify certain cultures that they encountered instead of trying to understand their complexity [16]. Because medical schools in Asia had few communication training programmes that focused on the Chinese-speaking context, students were prepared only to communicate medical information, not to address patients’ emotional needs [33].

The unique linguistic and sociocultural features of the Chinese-speaking context make Western teaching models inapplicable to such settings. This indicates the need for a new, culturally appropriate approach to clinical communication teaching in the Chinese-speaking context that is grounded on an understanding of the specific clinical needs of each field of medicine. Addressing this research gap can help build an effective system for teaching clinical communication in Chinese-speaking settings. Therefore, this study explored the perspectives of clinicians from different disciplines in Hong Kong to inform medical educators about current trends in teaching approaches, issues, and topics relevant to local clinical communication, with the aim of improving clinician–patient relationships.

While Hong Kong culture is similar to other Chinese cultures in some respects, Hong Kong also has distinctive cultural features that have implications for local clinical communication education. In one study, Hong Kong patients were found to be keen to appropriate the Western model of patient-centred care [28]. In another study, they were found to be open to discussions of advanced malignancy and willing to have direct involvement with their end-of-life arrangements [34]. Medical educators can address these patient preferences by helping students develop greater rapport and empathy with patients [35]. At the same time, such a teaching approach may counter the patriarchal elements of Chinese culture present in Hong Kong medical settings.

### Teaching clinical communication with a multidisciplinary approach

As confirmed by Tahir et al. in their investigation of interprofessional relationships and medical school teaching in primary social care settings [36–39], it is important to train clinicians in the early stages of their careers in clinical communication via approaches that integrate care strategies from different disciplines. Despite the established benefits of cross-disciplinary training, only a few empirical studies have gathered data from multiple disciplines to investigate doctor–patient encounters in hospitals, as noted by Jensen et al. in their own such study in Norway [40]. They found that the Four Habits training model (invest at the beginning, elicit the patient’s perspective, demonstrate empathy, and invest at the end), originally developed by Krupat et al. [41], was a suitable generic tool for teaching clinical communication to postgraduates across the following clinical settings (excluding psychiatry): anaesthesiology, paediatrics, surgery, internal medicine, gynaecology/obstetrics, neurology, orthopaedics, and ear–nose–throat medicine. They also found that a 20-hour intervention course derived from the Four Habits model was able to improve doctors’ communication skills and lead them to implement a more patient-centred model when treating patients. Their results show that through investigating the perspectives of clinicians from different disciplines, researchers may inform the development of medical communication training programmes that are applicable across many clinical settings. Such programmes may more effectively teach clinical communication, particularly in settings such as the intensive care unit (ICU) where physicians from different departments frequently cooperate with each other.

Extensive discipline-focused research on clinical communication teaching using interdisciplinary approaches has been conducted in the US [41] and European countries [36, 40]. However, no such studies have been conducted in Asian contexts, and in Chinese-speaking settings in particular. The importance of context-specific research that assesses the suitability of particular teaching approaches to clinical communication was illustrated by Bellier et al. [42]. Their cross-cultural study sought to explore the suitability of the Four Habits coding scheme for assessing clinical communication skills in France. According to their findings, the French version of the scheme demonstrated satisfactory internal consistency, but the real effects were moderate, and two raters were needed to effectively assess the clinical communication skills acquired by medical students. Therefore, it is necessary to conduct discipline-focused research on clinical communication teaching across specialties in different Asian and Chinese-speaking regions to gain a comprehensive overview of this area of medical education and to develop culturally appropriate, multidisciplinary teaching approaches.

This study focused specifically on the context of Hong Kong, as the city is considered an ‘entrepreneurial city’ in terms of its entrepreneurial discourse, narratives, and self-image [43]. Many sectors, including the medical sector and academia, adopt an entrepreneurial mindset in their daily operations in response to global trends in the knowledge economy [43]. Therefore, Hong Kong is a site at which the East converges with the West, and is a global hub for knowledge and cultural exchange. As a result, the city serves as an effective research base for researchers investigating intercultural clinical communication. Researchers may evaluate and compare Western and Chinese medicine in terms of clinicians’ communication strategies, patients’ expectations and satisfaction, and transfers between the two systems. Moreover, owing to the increasingly globalised patient population in Hong Kong, the city’s healthcare system exemplifies the general way in which healthcare is increasingly conducted in culturally and linguistically diverse settings around the world.

## Methods

### Research design

A cross-sectional, qualitative research design was used for this study. Specifically, our research team analysed interview data using interpretative phenomenological analysis (IPA), which originates from the field of psychology and focuses on the examination of personal lived experiences. According to Smith and Osborn [44], IPA is founded on individuals’ personal experiences and how they make sense of these experiences. Researchers who use IPA assume that individuals engage actively and continuously with their environments, following which they reflect on and integrate their personal experiences [44]. Therefore, IPA is fundamentally a type of allegorical analysis conducted by both the participants and the researchers involved. In conjunction with the participants, the researchers carry out a dual interpretation process, called the ‘double hermeneutic’, during which the researchers actively seek to determine how the participants engage in and make sense of their surrounding world [44]. In light of the diverse experiences of medical educators in teaching cultural competence, IPA was chosen as the analytical approach for this study because of its seminal nature and capacity to examine participants’ complex real-life lived experiences.

In addition, we used grounded theory to analyse the interview transcripts [45]. By approaching the data with as few preconceived theoretical notions as possible, we allowed themes to emerge from the analysis [46]. We searched and identified common threads in the transcripts that related to interdisciplinary communication and teaching approaches, thereby drawing out a wide range of views that captured the specific purposes of clinical communication teaching in the interviewed clinicians’ respective disciplines [47].

To arrive at a comprehensive overview of the current state of this teaching, and to contribute to the development of interdisciplinary teaching programmes for medical schools, we analysed the teaching approaches of experts from different disciplines. These disciplines were surgery, geriatrics, neurology, ICU medicine, nursing, oncology, palliative care, and obstetrics and gynaecology. We identified the different teaching strategies currently used and the communication skills considered to be important in each discipline.

Lastly, the methods and results are reported here according to the Consolidated Criteria for Reporting Qualitative Research (COREQ) guidelines [48].

### Data collection

Our research team conducted one-on-one, face-to-face semi-structured interviews with the participating clinicians in Hong Kong. These clinicians were from seven hospitals and two medical schools in Hong Kong. All of the participants were recruited via convenience sampling. They were proficient in speaking Cantonese, had been working for 9 years or more at a local hospital, and had earned a postgraduate degree in their specialty. Each interview were conducted in English and lasted 30–60 minutes, during which the team explored the clinician’s lived experiences, particularly in terms of the communication challenges encountered when interacting with their patients, patients’ families, and colleagues across disciplines, as well as in their teaching of clinical communication. For example, the clinicians were asked what effective clinical communication meant, how they communicated with their patients, carers, and colleagues from other disciplines to ensure that their explanations were understood, and what expectations they had for medical graduates regarding their communication skills.

### Participant demographics

Table 1 illustrates the participants’ demographic details. The participating clinicians’ (*n* = 9) clinical specialties spanned eight disciplines, namely surgery, geriatrics, neurology, critical care medicine in the ICU, nursing, oncology, palliative care, and obstetrics and gynaecology. Of the 9 participants, 5 were male and 4 were female. The participants had been working in clinical settings (either territory hospitals or teaching hospitals) for 9 to 13 years.

**Table 1:** Demographic details of the participating clinicians

Participant	Gender	Specialty	Work experience (years)	Clinical setting
A	M	Surgery	10	Territory hospital
B	F	Geriatrics	13	Teaching hospital
C	M	Neurology	13	Teaching hospital
D	F	Critical care medicine in the ICU	10	Teaching hospital
E	F	Nursing	14	Teaching hospital
F	M	Oncology	9	Territory hospital
G	M	Palliative care	11	Territory hospital
H	M	Surgery	11	Territory hospital
I	F	Obstetrics and gynaecology	13	Teaching hospital

### Analysis

Using IPA, an idiographic approach, we were able to analyse a tightly defined group of participants who drew from their lived experiences to express their opinions on the focal topic. Our research team comprised multidisciplinary experts (M = 3, F = 2), namely two linguists (PhD) who had extensive research experience in health communication, one physician (MD) from the accident and emergency department, one professor (PhD) in nursing, and one nursing manager (PhD). We adopted the suggestion of Smith et al. to analyse the interview transcripts in stages [47]. Following the IPA model, we first analysed the transcripts on an individual and descriptive level. Using these analyses, we then interpreted the data to draw out the teaching approaches of different medical disciplines. This process consisted of reading through the transcripts carefully line by line and noting down exploratory comments based on our interpretations of the participating clinicians’ use of language and teaching strategies. As noted by Smith et al. [49], such an approach, which uses the techniques of subsumption and abstraction, is helpful for constructing thematic analyses.

### Ethics

Written consent was obtained from all of the participants. The research was approved by the Human Ethical Review Committee of the City University of Hong Kong.

## Results

Five themes emerged from the thematic analysis. These were (1) empathising with patients; (2) using technology to teach both the medical and interpersonal aspects of clinical communication; (3) shared decision-making with patients and their families: the influence of Chinese collectivism and cultural attitudes towards death; (4) interdisciplinary communication between medical departments; and (5) the role of language in clinician–patient communication.

### Empathising with patients

Empathising with patients is the core element of patient-centred care. As discussed by Quince et al., clinicians who express empathy help their patients feel more satisfied, comfortable, and confident in their abilities, thus reassuring the patients [48]. Research has also shown that empathy can be a source of great emotional support for patients [28, 50], improve their treatment adherence, and encourage their more open disclosure of medical information [51, 52]. On the contrary, clinicians’ lack of empathy can lead to poor patient outcomes and patients’ distrust of clinicians [53–55]. All of these findings underscore the need for medical practitioners to be empathetic towards their patients. The following interview quotations reveal how the participants specialising in surgery, oncology, and nursing perceived this communication strategy.

#### Surgery

Surgeries are very important, sometimes life-saving, clinical procedures. Thus, it is important for surgeons to equip themselves with the communication skills of developing rapport and empathising with patients when delivering bad news. This communication strategy is not unlike that used in Chinese medical clinics, which emphasises patients’ emotional needs [30, 31]. Interviewee A noted the following:

*The most important thing is to have empathy. I think it really needs practice and also requires life experience in the outside world. You have to be engaged with the outside world.* — Interviewee A (Surgery)

As described by Interviewee A, surgeons tended to show their care by creating a space for patients’ families to tell their stories:

*If you are concerned about a patient, ask the patient’s family questions and make sure that your communication is not abrupt or brusque. In this way, they will sense that you care.* — Interviewee A (surgery)

Students must practise to develop the ability to engage in empathetic dialogue. Interviewee H noted that he usually instructed two students to act as the patient and the doctor, respectively. The roleplaying task would involve the following:

*[…] one tries to convey the bad news to the patient, and the patient tries to be a difficult patient, so [the patient] tries to act out, tries to be emotional and sees how [the doctor] cope[s] with that.* — Interviewee H (surgery)

#### Oncology

The interview data indicated that Interviewee F focused on their patients’ emotions. They stressed the following:

*You have to be ready to consider your clients’ emotions . . . [and] learn to provide reflective feedback [using] empathetic dialogu[e] and communication protocols.* — Interviewee F (oncology)

Interviewee F also stressed that oncologists should be constantly and actively aware of the relationship and degree of empathy they have with their patients:

*[It is] a balance of distance – you have to think [of] yourselves as both a healthcare provider and a friend of your client to bring your client closer; but sometimes when they ask for something that you cannot provide, you, in turn, will have to remind them of your role and your limitations.* — Interviewee F (oncology)

Accordingly, Interviewee F adopted the communication strategy of interspersing medical talk with interpersonal chat [35]. They mentioned that although doctors should show care towards their patients, they should also manage their time and be

*ready to interrupt gently by moving on according to your agenda when [pushed for time,] to make sure everything needed is covered.* — Interviewee F (oncology)

### Using technology to teach both the medical and interpersonal dimensions of clinical communication

Contemporary students have been exposed to technology since childhood [56, 57]. This has led medical educators to incorporate the use of technology into tertiary training programmes to teach clinical communication. Interviewee H (surgery) and Interviewee E (nursing) both confirmed this trend with regard to their experiences of teaching clinical communication. According to them, both surgeons and nurses at their hospital used video technology to educate their students on clinical communication. Senior surgeons and senior nurses used the technology in distinct ways to help students empathise with patients.

#### Surgery

Interviewee H believed that empathy constitutes a primary component of communication with the patient, mainly in the form of thinking from the perspective of others.

*I usually teach my students or the young doctors [the following:] pretend that your patient is your relativ[e] or even yourself and then try to think in their shoes and try to think what they are going through before actually starting a conversation with them or trying to [deliver] your bad news.* — Interviewee H (surgery)

One of the main ways in which medical students were taught the importance of empathy was through instructional videos. Interviewee H reported that senior medical staff from the surgery department screened instructional videos for their students that showed

*how [clinical communication] should be done, how we actually tell our patients when they have [a] terminal disease or so on, and also bad examples that [show how communication] may negatively influence the relationship between patients and doctors.* — Interviewee H (surgery)

By demonstrating how medical students should deal with difficult and emotional patients, these training practices prepared medical students for implementing the strategy of interspersing medical talk with interpersonal chat [35].

#### Nursing

Interviewee E said that she included students in the process of creating video content to promote healthcare information:

*We produced two sets of videos to try to help everybody in the local community […] enhance their health literacy about infection control measures.* — Interviewee E (nursing)

### Shared decision-making with patients and their families: The influence of Chinese collectivism and cultural attitudes towards death

In Hong Kong clinical settings, patients who have low levels of involvement in decision-making on their treatment often become dissatisfied with their healthcare providers [58]. This is because the local patient population overwhelmingly prefers healthcare models that involve patient participation [59]. For example, in Tam and Lau’s study of patient complaints in Hong Kong, almost half of the studied patients were concerned about their lack of communication with their clinicians [60]. These findings suggest that clinicians in Hong Kong should improve their clinical communication by being more attentive to their patients’ emotional needs and taking the latter’s opinions into consideration when making decisions on treatment. Through shared decision-making, clinicians can also ensure patients’ compliance with treatment plans [59]. However, although many recent studies have shown that Hong Kong patients desire greater participation in decision-making [35, 61], a very limited number of clinicians involve their patients in planning the course of their treatments, as demonstrated by Chandler et al. regarding communication in local accident and emergency departments (AEDs) [62]. As evident from the findings of the current study, local patients’ desire to participate in decision-making processes is not limited to AED settings. The interviewed geriatrics doctors, ICU-based critical care clinicians, and palliative care specialists all highlighted the need to include their patients throughout the process of making decisions on treatment.

#### Geriatrics

Considering the age of patients in the geriatrics department, the goal of clinicians in this department, according to Interviewee B, was

*not necessarily to care for our patients in terms of curing them of their medical problems; a lot of times our patients have illnesses that can’t be cured.* — Interviewee B (geriatrics)

Because the goal of patient care in these cases was not always to cure patients’ illnesses, geriatricians had to adopt the communication strategy of creating space for patients to tell their stories [35]. Offering such a space facilitated the shared decision-making process as it helped clinicians understand patients’ needs and provide treatment that aligned with what the latter valued most. Interviewee B further elaborated that the clinician team would then negotiate decision-making on treatment with the patients and their families [35], arriving at

*a mutual understanding [with patients and their families] of what a treatment is able to achieve and what it is not able to achieve.* — Interviewee B (geriatrics)

#### ICU-based critical care

Similarly, the communication strategy of shared decision-making [35] on treatment was important in the ICU, as it helped

*bring all parties together to formulate a plan that’s best for the patient.* — Interviewee D (ICU)

Moreover, because of the complexity of the patients’ diseases, their families often found it difficult to digest all of the relevant medical information. Therefore, ICU doctors found themselves

*repeating similar information over the course of a few days; only then does the information start to sink in and relatives start to understand and come on board with us […] in terms of the plan for management.* — Interviewee D (ICU)

ICU doctors also adopted approaches that were inclusive of the patient [35] when making decisions, because

*maybe more often than you think, our patients are actually conscious, and it helps to conduct the interview in the presence of the patient as well.* — Interviewee D (ICU)

#### Palliative care

Interviewee G added that family culture and beliefs regarding death also had to be taken into consideration when negotiating the direction of treatment. Family dynamics played an important role in the process of decision-making, especially in end-of-life situations. As stated by Interviewee G,

*one must be proactive in preparing carers’ expectations along the course of the disease. They have to be told what to be expect, such as the gradual deterioration of the patient, and how to act. When, for example, breathing is becoming difficult, they have to call the ambulance. They have to be told what to discuss with the patient, such as their will, last wishes, advanced directives, and the aftermath. All of this has to be done tactfully over time and by developing rapport. Essential family dynamics must also be observed, as well as family culture regarding death and dying and suffering.* — Interviewee G (palliative care)

Therefore, patients’ families were an important stakeholder in healthcare communication. End-of-life situations had to be dealt with carefully, because

*families can help to ease patients’ suffering but can also pose a huge problem [if communication about treatments is not well-handled].* — Interviewee G (palliative care)

Overall, the above data show that the communication strategy of shared decision-making on treatment was particularly important to the field of palliative care.

### Interdisciplinary communication between medical departments

As discussed by Bok et al. [63], effective communication between healthcare professionals from various disciplines helps facilitate negotiation and shared decision-making [64]. The strategy of shared decision-making is thus not limited to clinicians’ interactions with patients and their families; it is also applicable to interactions between clinicians from different disciplines and between clinicians and external supporting parties. Both the neurology specialist and the intensive care specialist interviewed in this study expressed the belief that medical students should learn how to collaborate with clinicians from other disciplines.

#### Neurology

After delivering bad news to patients and their family members, neurologists could support them by collaborating with

*nurses, allied health workers, psychologists, social workers.* — Interviewee C (neurology)

##### ICU-based critical care

ICU work was primarily interdisciplinary, because

*in critical illness often more than one organ system is involved, so it really takes a lot of time and repeated interviews to try to get the patient to understand what is happening to them and to have a realistic expectation of how things will progress.* — Interviewee D (ICU)

This process involved a lot of communication between various medical disciplines, as patients

*might have problems simultaneously with their heart, with their lungs, their kidneys, these are major organs that are involved in an acute illness . . . [and the patient has to] be in close communication with a cardiologist, respirologist [or] surgeon if an operation is planned or if the patient is in the early post-operative stage. And then we also speak with dieticians to try to optimise nutrition. We have to speak to physiotherapists and speech therapists and come up with a plan for management as a whole team.* — Interviewee D (ICU)

### The role of language in clinician–patient communication

In the study of Pun et al. on Hong Kong clinicians’ perceptions of communication challenges in the trilingual environment of a local emergency department, the clinicians felt that they were unable to engage in interpersonal communication with their patients because of linguistic complexity, long working hours, time constraints, and high patient loads [32]. Although most of those respondents recognised patient-centred care to be optimal, they rarely listened to their patients [28] and prioritised basic duties over developing empathy or rapport with their patients through interpersonal communication [35]. Moreover, as mentioned in the Background section above, Hong Kong clinicians mainly speak with patients in their mother tongue, Cantonese, but receive their medical school training in English [31]. Because of the large number of immigrants from mainland China, they also speak with some patients in Mandarin, which makes local clinical settings trilingual and linguistically complex [65]. Language plays an important role in clinicians’ attempts to intersperse medical talk with interpersonal chat with patients, because this strategy requires the clinicians to translate English medical jargon into plain language in Cantonese under the high pressure of Hong Kong’s clinical environments [35]. In this study, the participants working in the disciplines of neurology, intensive care, and nursing said that they addressed this challenge by shifting between technical and conversational language to explain medical concepts clearly, repeating key information, and communicating through both verbal and non-verbal means [35]. Researchers in other countries may also find our findings useful, as these may help them become aware of the linguistic complexity and conditions in their own countries, and of how patient–provider interaction and communicative understanding may be accordingly affected.

#### Neurology

To clearly describe diseases and medical concepts to patients and their families, neurologists at Interviewee C’s hospital moved between technical and conversational language [35]. Interviewee C noted that it is essential

*to break down messages in small chunks, into very very simple messages, and then ask the translator to tell them or to back-translate.* — Interviewee C (neurology)

Such a communication strategy required neurologists

*to use as little medical jargon and complex terminology as possible, and to use simple sentences and simple phrases.* — Interviewee C (neurology)

Interviewee C, for instance, used analogies or examples

*day in and day out […] I use examples to convey complex problems to our patients and caregivers, so they can relate to what is going on.* — Interviewee C (neurology)

For example, Interviewee C often compared the functioning of the heart to the mechanism of a kitchen tool:

*It is like your blender in your kitchen. If you press different buttons to change the speed of the blender, 0 1 2 3 4, it will not blend nicely and it will form curves, so on and so on, and if you imagine this is the heart, if it is not beating regularly, then clots can form, but then obviously if these block any blood vessels, then there can be detrimental consequences.* — Interviewee C (neurology)

By making medical knowledge accessible, the neurologists at Interviewee C’s hospital put themselves in the shoes of the patients and their families, thereby making clinical interactions

*a two-way process [of] mutual respect.* — Interviewee C (neurology)

#### ICU-based critical care

Due to the complexity of patients’ diseases in the ICU, it was hard for patients’ families to digest all of the medical information being communicated to them. Therefore, ICU doctors found themselves

*repeating similar information over the course of a few days; only then does the information start to sink in and relatives start to understand and come on board with us […] in terms of the plan for management.* — Interviewee D (ICU)

In addition to this verbal communication strategy of repeating key information, ICU doctors also used non-verbal resources:

*[We] give them some information, leaflets to take home or some resources to go back to that helps them digest the information we are trying to convey.* — Interviewee D (ICU)

#### Nursing

To meet the needs of patients with disabilities such as hearing loss, Interviewee E used non-verbal languages such as sign language. For example, she and her students produced two sets of videos, the objective of which was

*to try to help everybody in the local community. […]We added a little square for one of my students who knew sign language.* — Interviewee E (nursing)

## Discussion

As stated in the Results section, the five themes that emerged from the thematic analysis were as follows: (1) empathising with patients; (2) using technology to teach both the medical and interpersonal dimensions of clinical communication; (3) shared decision-making with patients and their families: the influence of Chinese collectivism and attitudes towards death; (4) interdisciplinary communication between medical departments; and (5) the role of language in clinician–patient communication.

Clinical communication teaching can have a significant effect on the development of medical students’ communication skills, which are important for their communication with colleagues, patients, and patients’ families. It is particularly important to consider the types of teaching approaches used when designing medical education programmes [29]. To implement patient-centred care, it is important for medical students specialising in different disciplines to equip themselves with varied communication strategies suitable for specific cultural contexts. For example, as concluded by Pun et al. in their literature review [28], while Confucian values in mainland China have led medical students there to follow a more clinician-led model [65], students in Hong Kong should adopt communication strategies that increase patients’ level of involvement in decision-making. Therefore, by drawing out themes from our interview data in this Hong Kong-based study, we laid a foundation for the development of culturally appropriate communication training for medical students across disciplines. The study contributes to the exploration of interdisciplinary clinical communication strategies and teaching approaches in multilingual and multicultural contexts, and facilitates the development of frameworks that enhance interdisciplinary clinical interactions to meet the different communication needs of patients from a wide range of cultural backgrounds.

### Empathising with patients

The clinicians we interviewed from the disciplines of surgery, nursing, and oncology all noted the importance of empathising with patients as a component of clinician–patient relationship building. In addition, they all described teaching approaches to encourage students to engage in empathetic conversations with patients. This finding echoes the results of Slade et al. [35], who found that surgeons and oncologists in particular stressed the importance of having interpersonal chats in addition to transactional, medical talk with patients (e.g., talking about diagnoses, symptoms, and treatment). Another study found that a lack of empathy could result in poor patient–provider relationships, thus supporting our interviewees’ stance [66]. The observations of the clinicians in this study echoed recent research on the topic that has found a causal relationship between clinical communication skills and medical outcomes. Oncologists’ communication skills, for example, have been found to have a large impact on patients’ satisfaction, health outcomes and compliance with treatment [67, 68].

Their teaching approaches indicate their belief that medical students should focus on not only their patients’ physical needs, but also their emotional needs. This belief is supported by Slade et al., who found that combining medical talk with interpersonal conversations improved patients’ evaluations of the quality of their care and increased their involvement in decision-making on their treatment [35]. However, while engaging in empathetic and relational talk is an important way to improve patient care, the data from the current study also suggest that medical students have to be strategic in their use of this communication approach, as engaging in interpersonal conversations may encroach on the time allocated for treatment.

The stressful environment in Hong Kong hospitals makes it difficult for nurses to meet patients’ holistic needs [69], i.e., their spiritual, psychological, and emotional needs in addition to their physical needs [70]. One study suggested that Chinese nurses neglected patients’ emotional support and instead prioritised their physical comfort, due to the family-centredness of Chinese culture and the exclusion of nurses from challenging end-of-life conversations. The excluded nurses became hesitant to talk about terminal illnesses with dying cancer patients, as death is a taboo subject in traditional Chinese culture, which made it hard for the nurses to provide these patients with emotional support [28]. However, Interviewee E (nursing) noted the necessity for nurses to attend to patients’ emotional needs. Our interviewee’s stance coincides with the findings of Liu et al. [71], who found that Chinese cancer patients valued their nurses’ emotional support and caring behaviours despite the commonly accepted cultural preference in Chinese-speaking clinical settings. In addition, nurses play a central role in the delivery of culturally competent healthcare services, especially to patients affected by linguistic and cultural barriers in Chinese-speaking contexts [72]. Therefore, it is necessary to help nursing students develop empathy with their patients, especially as the scope of real-life nursing practice includes patient advocacy, education, and the promotion of health [73, 74].

This pedagogical need is especially important for cases in which patients have disabilities such as deafness, which makes linguistic barriers especially difficult to surmount. To guarantee equal access to healthcare information and services for such patients, educators should help nursing students gain experience in communicating with these patients. The findings of the aforementioned studies highlight the need to review the current approach to nurse–patient relationships. Our interviewee from the discipline of nursing, Interviewee E, focused especially on the concerns of patients with special needs in their interview. She described how senior nurses in Hong Kong clinical settings tended to train their students to attend to patients with special needs. This was done, for example, by involving students in the processes of helping patients with special needs, so that the students could learn the relevant medical information. She cited the specific instance of creating instructional healthcare videos with her students, in relation to which she emphasised the importance of empathetically bearing in mind the needs of patients with hearing loss. The interviewee also noted that the educational process she described could raise the students’ awareness about disabilities and make them more empathetic towards disabled patients.

The findings of this study indicate that the manner in which empathy is shown is also subject to influence by cultural factors in clinical communication. While it is obvious that empathy should be shown for everyone, being empathetic towards patients’ families was particularly emphasised in the context of this study. The importance of the family in Asian clinical settings was highlighted by Ishikawa and Yamazaki, who observed that within Asian cultures, patients are treated not as individual units but as parts of larger social units, particularly in the context of decision-making [14]. Therefore, Western individualistic models are inapplicable to Asian contexts, and sociocultural differences should be taken into consideration when conducting communication in different cultural contexts. For example, the interviewed surgeons in our study highlighted the need for medical students in Hong Kong to ask patients’ families questions and express care for them, thereby giving the families the space to tell their stories. By adopting such a strategy, students would be able to empathise with patients’ families, thus not only helping to improve levels of quality care and patient satisfaction, but also facilitating shared decision-making processes. These insights underscore the need for medical education programmes to include the teaching of communication skills and cultural elements, focusing in particular on empathy. To this end, the design of clinical communication training sessions should align with students’ interests and current trends.

### Using technology to teach both the medical and interpersonal dimensions of clinical communication

One of the surgeons we interviewed, Interviewee H, mentioned the use of videos to teach clinical communication. According to this interviewee, videos are effective means of showing medical students how to communicate with patients about terminal illnesses, as well as how not to do so. These findings indicate the feasibility of medical students using videos as instructional materials to acquire clinical communication skills. Many studies have shown that because students find videos particularly useful for revision and preparation for medical practice, video instruction can help ensure performance outcomes and boost students’ confidence [75–77]. As a result, students prefer instructional videos to conventional face-to-face instruction [76, 77]. Videos are also easily accessible because of the ubiquity of portable video-playing devices such as smartphones [78].

Most of these studies, however, have been conducted in English-speaking countries and have not focused on the teaching of clinical communication. In this study, we noted this research gap and sought feedback on the suitability of using videos for teaching clinical communication in Hong Kong. Interviewee H confirmed that instructional videos showcasing scenarios in Hong Kong clinical settings could help surgical students prepare for interspersing medical talk with interpersonal chat with patients [35].

Interviewee E (nursing) further described a different approach to using video technology in clinical communication teaching. She described how senior nurses involved students in the process of creating healthcare-related video content. This approach of using videos as a teaching tool was consistent with current trends in Asian nursing education: for example, an interviewed nurse supervisor who participated in Huang’s study had also received extensive training on the latest technology in nursing care [69]. Through their approach, the nurses described by Interviewee E encouraged their students to think of unconventional ways of communicating with patients. These nurses also helped their students understand how technological advances have facilitated interactions between nurses and patients, particularly disabled patients with linguistic barriers [72]. Teaching with video may also be an inclusive approach that takes into consideration students’ interests, as technological devices are a ubiquitous feature of nursing students’ lives nowadays, as suggested by Kelly et al. [79]. Moreover, medical educators could use digital media to inform students on how information technology could be used to deliver healthcare information to the public beyond patients. Medical educators could thus encourage more creative uses of evolving digital technologies in medical settings by involving students in the process of creating relevant content. Building on their other learning experiences, students could learn to increase patient engagement through technological means and explore how health information technology could create new modes of media interaction between patients and clinicians [80].

### Shared decision-making with patients and their families: The influence of Chinese collectivism and cultural attitudes towards death

In this study, the interviewees working in geriatrics, ICU-based critical care, and palliative care noted the communication gap between most clinicians and their patients. As a result, they deemed it necessary to teach their students how to develop strategies for shared decision-making with patients and their families.

One of the strategies mentioned by Interviewee B, a geriatrics doctor, was creating space for patients to tell their stories [35]. For example, this was done was by asking them what their goals were and what ‘they value[d] the most’, before coming to a ‘mutual understanding of what a treatment [would be] able to achieve’ with the patients. Interviewee B noted that in light of the incurability of many illnesses, medical students must understand their patients’ goals when participating in decision-making on treatments and plans with patients. The interviewee thus found it particularly important for clinicians, patients, and their families to come to a consensus regarding the benefits and limitations of certain treatments and which choices were best aligned with the patients’ interests during the shared decision-making process [35].

The interviewed ICU clinician, Interviewee D, also noted that they took an inclusive approach to decision-making, even with patients who could not speak. It was important for the interviewee to conduct interviews in front of these patients because they believed that in many cases, the patients – such as those who were intubated – were actually conscious and desired to actively participate in medical discussions. Leung et al. support this belief [81].

Moreover, given the collectivist attitude promoted by Chinese culture and other Asian culture, such as highlighted by Ishikawa and Yamazaki [14], clinicians usually advise their students to involve patients’ families in shared decision-making on treatment [35]. In this study, this involvement was mentioned by the interviewed ICU clinicians and palliative care specialists. In many East Asian cultures, there is a three-way clinician–patient–family dynamic [82, 83], and decision-making is family-centred [84]. For the interviewed ICU clinician, it was important to ensure that patients’ families understood different treatment options and agreed with the suggested decisions, which could be facilitated by the strategy of repeating key information [35]. Information was often repeated in both verbal and written forms such as interviews and leaflets. The interviewed palliative care specialist, Interviewee G, further discussed the importance of observing family dynamics when engaging in end-of-life communication, through which clinicians had to manage the expectations of patients’ families such as those pertaining to the patients’ deterioration, measures to take in emergencies, and the patients’ last wishes. The interviewee emphasised that it was important for students working on such cases to learn about the family’s dynamics and attitudes towards death. In these end-of-life cases, clinical communication is highly dependent on the context, especially in light of Chinese cultural taboos regarding the discussion of death [28]. Therefore, medical educators in Chinese contexts should teach students not to engage in explicit conversation about death in end-of-life scenarios, but rather attune to the high-context nature of such scenarios and use indirect expressions or non-verbal communication [85].

### Interdisciplinary communication between medical departments

Learning how to negotiate during interprofessional shared decision-making is important mainly due to the interdisciplinary nature of most medical procedures. For example, the interviewed neurologist, Interviewee C, described the need in their discipline to offer emotional and physical support to patients and their families through working with nurses, allied health workers, psychologists, and social workers. The interviewed ICU clinician, Interviewee D, also reported their experiences of negotiating treatment options with other departments [35]. For instance, they would discuss their patients’ conditions with clinicians from other departments to negotiate and arrive at the best treatment plan. They also stated that this was often necessary because patients tended to have more than one damaged organ, thus requiring the care of other specialists such as surgeons and cardiologists. However, despite the benefits of interdepartmental collaboration evident from our study, Ng et al. reported that many of their interviewees found the culture of speaking up to be underdeveloped in interdepartmental communication, because of hierarchies between clinical departments [86].

Although some existing programmes aim to help medical students develop the skills for engaging in effective interdisciplinary communication, most programme designs lack a longitudinal perspective and the effective means to appraise competency [63]. Therefore, it is important for medical educators to consider recent studies and cultural contexts when designing interprofessional communication training programmes that aim to help students recognise the interwoven nature of cognitive and procedural knowledge across different clinical settings [63]. Such training should make the process of shared decision-making smoother across disciplines and allow for better cohesion and interdisciplinary communication.

### The role of language in clinician–patient communication

In relation to the final theme, Interviewee C, a neurologist, described the way in which he encouraged students to adopt the communication strategy of explaining medical concepts clearly by shifting between technical and plain language [35]. To do so, students had to first break down medical information ‘into small chunks’ to convey ‘simple messages’, while avoiding excessive use of English ‘medical jargon and complex terminology’, then pass these messages on to the translator. In addition, Interviewee C gave examples of interspersing medical talk with interpersonal chat with patients [35]. One strategy he mentioned was to ‘convey complex problems’ to patients using everyday examples, such as by comparing the mechanism of the heart to the motion of a blender. By shifting between technical and plain language and interspersing medical talk with interpersonal talk, clinicians would be able to communicate medical knowledge to patients and thereby develop good clinician–patient relationships [35].

To make sure that they clearly conveyed medical knowledge to patients and their families, some clinicians were also reported to use the strategy of repeating key information [35]. For instance, Interviewee D, an ICU-based critical care physician, noted that it was sometimes important to repeat ‘similar information’ to patients’ family members until they complied with treatment plans. This particular strategy, along with the delivery of additional information in written form, was identified as being able to help patients’ families understand the different treatment options and their respective benefits and drawbacks. Moreover, by couching this information in plain language, clinicians could help patients’ families more easily digest the medical information before making shared decisions.

Another point that we have not fully expanded upon due to limited data, but nonetheless warrants further research, is the role and function of non-verbal languages. Especially in clinical settings, some patients have disabilities such as hearing loss. It has been found that many deaf patients experience medicine-related mistreatment because of ineffective communication and lack of access to information [87]. Such scenarios fall short of the standards described in the United Nations’ Disability Rights Agreement, which advocates equal access to healthcare services for the disabled [88]. Interviewee E, a nurse, noted this issue and described how nursing students were involved in the process of creating media content that had sign language subtitles. Such an inclusive approach to treating deaf patients runs counter to the Chinese cultural norm, whereby a functional approach is adopted to meet patients’ medical and emotional needs [71, 89]. The disability barrier example raised by Interviewee E indicates the need for medical students to consider ways of communicating medical knowledge with patients with disabilities, such as by asking medically experienced sign language interpreters for help. To implement patient-centred care, it is necessary to take patients with special needs into consideration.

### Limitations

A general limitation of this study is that its findings were derived from a small data sample of nine participants. This might have skewed the representativeness of the themes, thus challenging the reliability and validity of the findings. Moreover, despite the extensiveness of the research process, which consisted of the IPA methodological process, the formulation of themes, and the categorisation of data extracts under the corresponding theme, the interview data were subject to interpretation and researcher bias. Future research to support, counterargue, or build upon the study’s findings is warranted. In addition, due to the complexity and richness of the data, the themes could have overlapped on similar points and consequently become difficult to demarcate. For example, although we designated ‘empathising with patients’ and ‘the role of language in clinician–patient communication’ as two separate themes, a main way in which medical professionals might have shown empathy would have been through the use of language.

## Conclusions

While medical education programmes in Western countries have benefited from research, testing, and innovative reforms over the decades [33], those in Asian regions have lagged behind. To implement effective patient-centred care in Asian cultural contexts, medical educators must train medical students to be culturally competent. Therefore, through examining Hong Kong clinicians’ observations, opinions, teaching approaches, and experiences across disciplines with regard to clinical communication teaching, this study suggested possible approaches to such teaching according to specific cultural values. As most studies on clinical communication teaching have been conducted in Western settings, this study also adds to the literature in the form of an Eastern case study. It helps to remedy the possible neglect of Eastern developments in the literature and presents findings that are more applicable to Eastern settings. Our suggestions may encourage future designers of medical education programmes to focus on developing students’ communication skills and cultural competence through implementing local adaptations of patient-centred models.

By adapting clinical communication to fit East Asian contexts such as Hong Kong, researchers and clinicians may be able to move beyond the arguably cumbersome East–West comparison and focus on more specific geographical and sociocultural contexts. The well-being of patients is affected by several factors, such as their medical problem (which determines the relevant medical discipline), background, culture, environment, beliefs, and even clinicians’ experiences. Considering the cultural diversity of Hong Kong, it is crucial for clinicians to consider how the definition of well-being might vary for patients depending on their cultural backgrounds and beliefs at the micro level. For instance, as discussed in this study, patients from Asian backgrounds tend to see themselves as part of a bigger social unit, as Asian cultures advocate a family-centred model that emphasises the value of family decision-making processes. On the contrary, those from or educated in Western contexts tend to be more aware of their individual needs and rights when making decisions on their treatments [84]. Given the plethora of factors affecting patients’ well-being, clinical communication must always be adapted to be sensitive and attuned to patients’ perspectives, cognition, and milieu, as different patients have different communication needs.

This study is one of the very few studies in Hong Kong to provide a comprehensive account of clinical communication across multiple disciplines in local hospitals, and to advocate the teaching of interdisciplinary communication to more effectively facilitate shared decision-making among different specialists. The findings of this study indicate the value of conducting region-wide quantitative and interdisciplinary research on clinical communication, and may guide future researchers based in Asia. However, due to the small sample size of our study, generalisations of the findings should be made with caution.

## Data Availability

All data generated or analysed during this study are included in this published article.
